# Novel *KCNJ10* Compound Heterozygous Mutations Causing EAST/SeSAME-Like Syndrome Compromise Potassium Channel Function

**DOI:** 10.3389/fgene.2019.00912

**Published:** 2019-11-08

**Authors:** Hongfeng Zhang, Lin Zhu, Fengpeng Wang, Ruimin Wang, Yujuan Hong, Yangqin Chen, Bin Zhu, Yue Gao, Hong Luo, Xian Zhang, Hao Sun, Ying Zhou, Yi Yao, Xin Wang

**Affiliations:** ^1^Fujian Provincial Key Laboratory of Neurodegenerative Disease and Aging Research, Institute of Neuroscience, School of Medicine, Xiamen University, Xiamen, China; ^2^Department of Functional Neurosurgery, Xiamen Humanity Hospital, Xiamen, China; ^3^Departments of Neurosurgery, Dongfang Affliated Hospital of Xiamen University, Xiamen, China; ^4^National Institute for Data Science in Health and Medicine, School of Medicine, Xiamen University, Xiamen, China; ^5^Department of Neurosurgery, Shenzhen Children’s Hospital, Shenzhen, China

**Keywords:** KCNJ10, Kir4.1, K^+^ channel, compound heterozygous mutations, SeSAME/EAST syndrome

## Abstract

Inwardly rectifying K^+^ channel 4.1 (Kir4.1), encoded by *KCNJ10*, is a member of the inwardly rectifying potassium channel family. In the brain, Kir4.1 is predominant in astrocytic glia and accounts for the spatial buffering of K^+^ released by neurons during action potential propagation. A number of studies have shown that mutations in *KCNJ10* are associated with SeSAME/EAST syndrome, which is characterized by seizures, ataxia, sensorineural deafness, and electrolyte imbalance. Herein, we identified two siblings presenting with seizures and motor delays in one outbred kindred. Customized targeted-exome sequencing showed that both affected siblings are compound heterozygous for two *KCNJ10* missense mutations (NM_002241.4: c.601G > A: p.A201T and c.626T > C: p.I209T). Prediction tools suggested that both amino acid substitutions were deleterious or disease causing. Further functional studies showed that Chinese hamster ovary (CHO) cells expressing either A201T and/or I209T Kir4.1 channels exhibited lower K^+^ currents, indicating compromised Kir4.1 biological function. Intriguingly, the A201T but not I209T mutation decreased total and cell surface Kir4.1 levels. Kir4.1 channels with the A201T mutation were unstable and degraded through lysosomal pathway. In conclusion, these data indicated that both A201T and I209T mutations disrupt Kir4.1 activity and are the cause of SeSAME/EAST-like syndrome in the siblings.

## Introduction

Inwardly rectifying K^+^ channel 4.1 (Kir4.1), a member of the inwardly rectifying K^+^ (Kir) channel family, is predominantly expressed in glial cells of the brain (astrocytes and oligodendrocytes) (Takumi et al., 1995; Seifert et al., 2009), inner ear (Takeuchi et al., 2001; Ishii et al., 2003), and kidney (Garcia et al., 2007). In various cell types of the inner ear, Kir4.1 regulates K^+^ homeostasis and is involved in endocochlear potential generation and maintenance, which is essential for cochlear development and hearing (Chen and Zhao, 2014). In the basolateral membrane of the distal convoluted tubules (DCTs), Kir4.1 contributes to K^+^ recycling and generation of a negative membrane potential (Zhang et al., 2014; Su and Wang, 2016). Glial Kir4.1 channels account for extracellular K^+^ buffering, glutamate uptake, astrocyte development, and myelination (Neusch et al., 2001; Kucheryavykh et al., 2007; Djukic et al., 2007).

Autosomal recessive mutations in the *KCNJ10* gene cause a multisystemic disorder termed SeSAME/EAST syndrome, which is characterized by seizures, ataxia, sensorineural deafness, electrolyte imbalance, and developmental delay (Bockenhauer et al., 2009; Scholl et al., 2009; Reichold et al., 2010). To date, approximately 20 different pathogenic variations of *KCNJ10* have been reported. Most of the patients harbor homozygous or compound heterozygous mutations, while other types of mutations (e.g., nonsense) are rare (Bockenhauer et al., 2009; Scholl et al., 2009; Reichold et al., 2010; Freudenthal et al., 2011; Scholl et al., 2012; Kara et al., 2013; Papavasiliou et al., 2017;Abdelhadi et al., 2016;Al Dhaibani et al., 2018;Nicita et al., 2018). Herein, we report two novel variants of *KCNJ10 *in a compound heterozygous state in two siblings who manifest epilepsy and motor delays, two cardinal symptoms of SeSAME/EAST syndrome. Functional analysis *in vitro* showed that both variants disrupt Kir4.1 channels function, indicating that both mutations are pathogenic. We conclude that the novel compound heterozygous mutations in *KCNJ10* are likely responsible for SeSAME/EAST-like syndrome in the two siblings.

## Materials and Methods

### Patients

The two patients are siblings. The elder sister is 3 years old, and she has suffered from epilepsy since the age of 7 months. The younger brother is 1 year 8 months old. He has had epilepsy since the age of 6 months, and the seizure semiology was similar to that of his elder sister. Their parents were healthy, and their grandfather had a cerebral contusion at 40 years old and has had secondary epilepsy since then.

### Molecular Genetic Analyses

Genomic DNA was extracted from peripheral blood samples from the two patients and their parents using standard protocol. DNA libraries were prepared using a Joy Orient DNA Library Preparation Kit (Joy Orient Translational Medicine Research Center Co. Ltd., Beijing, China), in which platform-specific adaptors and unique DNA indexes are ligated. The libraries were tested for enrichment by quantitative polymerase chain reaction (qPCR) and for size distribution and concentration using an Agilent Bioanalyzer 2100 (Agilent Technologies, USA). Targeted next-generation sequencing was performed using a SeqCap Clinical Exome sequencing panel (Roche AG., Basel, Switzerland) customized by Joy Orient, which targeted 3,372 genes that are potentially associated with 4,213 known diseases with Mendelian inheritance by capturing 7,465,978 bp of targeted exon regions using 91,867 probes. A HiSeq 2500 sequencer was used to sequence the samples as instructed by protocols (version 3, Illumina, Inc., San Diego, California). Raw image files were processed by the BclToFastq (Illumina) for base calling and generating the raw data. The low-quality variations were filtered out using the quality score ≥ 20 (Q20). The sequencing reads were aligned to the National Center for Biotechnology Information (NCBI) human reference genome version hg19 using BWA. Samtools and Pindel were used to screen single-nucleotide polymorphism (SNP), insertion, and deletion mutations of the sequence. All genetic variants were screened by pathogenicity, mode of inheritance, and clinical phenotypes, and we identified two variants, c.601G > A and c.626T > C, in the *KCNJ10* alleles, respectively, that are potential disease causing in the patients.

To predict the possible impact of missense mutations on the structure and function of Kir4.1, four bioinformatic tools were used: PolyPhen-2 (Adzhubei et al., 2010) (polymorphism phenotyping, v2), PROVEAN (Choi and Chan, 2015) (protein variation effect analyzer, v1.1.3), SIFT (Kumar et al., 2009), and MutationTaster (Schwarz et al., 2014). The degree of amino acid conservation of KCNJ10 was evaluated with Clustal Omega.

### Construction of Wild-Type and Mutant KCNJ10 Plasmids

Human *KCNJ10* cDNA was cloned, fused with a human influenza hemagglutinin (HA) tag on its N terminus and inserted into a pCDH-CMV-MCS-EF1-copGFP vector (System Biosciences, CD511B-1). This KCNJ10-HA served as a template into which the c.601G > A or c.626T > C variant was introduced using a KOD-plus-mutagenesis kit (SMK-101, TOYOBO). The integrity of all the constructs was verified by Sanger sequencing.

### Electrophysiology

Chinese hamster ovary (CHO) cells were plated in poly-d-lysine-coated sterile glass coverslips and cultured in Dulbecco’s modified Eagle medium (DMEM)/F-12 (Gibco, 11320033) with 10% fetal bovine serum (FBS) (FS101-02, TransGen Biotech, China). After 60% confluence was reached, CHO cells were transfected with the wild-type (WT), c.601G > A, and c.626T > C KCNJ10 plasmids or co-transfected with WT and c.601G > A, WT and c.626T > C, and c.601G > A and c.626T > C plasmids using TurboFect transfection reagents. Cell transfected with empty vector (mock transfected) served as a control. Twelve hours after transfection, patch clamp experiments were conducted on fluorescing cells under ultraviolet light. Typically, we used a bathing solution (containing (mM) 40 KCl, 100 NaCl, 1.8 CaCl_2_, 0.53 MgCl_2_, 5.5 glucose, and 5.5 HEPES-KOH (pH 7.4)) and an intracellular solution [containing (mM) 140 KCl, 5 K_2_ ATP, 1 MgCl_2_, 5 EGTA, and 5 HEPES-KOH (pH 7.3)]. Currents were recorded with voltage steps from −120 to +40 mV in 10-mV steps, with a holding potential of 0 mV. Current signals were amplified using a Multiclamp 700B amplifier (Axon Instruments), digitized using a 1440A digidata, and recorded with Clampex 10.6. Data were analyzed using MiniAnalysis (Synaptosoft). Each average steady-state current was measured at the end of 300 ms during the voltage step-pulses duration.

### Cell Surface Protein Biotinylation

293T cells plated in poly-d-lysine-coated dishes (60 mm) were transfected at 70% confluence with 6 μg of WT or mutant *KCNJ10* plasmids. Twenty-four hours later, cell surface protein biotinylation was performed using a commercial biotinylation kit. Briefly, cells were incubated with Sulfo-NHS-LC-biotin (Thermo Scientific, 21335) (0.5 mg/ml) for 20 min at 4°C. The reaction was quenched, and the cells were scraped for lysis. Following cell lysis, the protein supernatant was collected and then incubated with immobilized streptavidin agarose (Thermo Scientific, 20347) overnight at 4°C. Biotinylated proteins were eluted with sample buffer and separated by sodium dodecyl sulfate–polyacrylamide gel electrophoresis (SDS-PAGE), followed by western blot analysis. Nonbiotinylated samples were used as a negative control (input).

### Quantitative Real-Time PCR

293T cells plated in six-well cell culture plates were transfected with the empty vector, WT, or mutant KCNJ10 plasmids for 24 h. Total mRNA was extracted using TRIzol reagent according to the manufacturer’s instructions and then reverse transcribed to cDNA by a PrimeScript reverse transcription reagent kit (TaKaRa, RR047A). The expression of KCNJ10 was detected by FastStart Universal SYBR Green Master (Rox) (Roche, 04913914001) on a LightCycler^®^ 480 System (Roche). Primers for KCNJ10-HA were forward 5′-CTGAAAAGCTCAAGTTGGAGGA-3′, reverse 5′-GTAATCTGGAACATCGTATGGGTAG-3′; primers for internal control ACTB were 5′-TGACGTGGACATCCGCAAAG-3′, reverse 5′-CTGGAAGGTGGACAGCGAGG-3′. All samples were assayed in triplicate in an optical 96-well reaction plate. Data analysis was performed by the comparative Ct method, and β-actin served as the reference gene. The results were expressed as the ratio of Kir4.1-HA mRNA level to β-actin mRNA level.

### Pharmacological Treatments of Proteasomal and Lysosomal Inhibitors

293T cells transfected with *WT* or *601G > A* *KCNJ10* plasmids were incubated for 6 h with 0.1% dimethylsulfoxide (DMSO), lysosomal inhibitors (100 μg/ml leupeptin (Sigma, 62070) and 50 mM of NH_4Cl_) or proteasomal inhibitors [10 μM of MG132 (MCE, HY-13259)]. After treatment, cells were lysed and subjected to western blot analysis.

### Statistics

Statistical analyses were performed with GraphPad Prism 6 (GraphPad Software Inc., La Jolla, USA). Data distribution was assessed by a Kolmogorov–Smirnov nonparametric test of equality. Differences among multiple means were assessed, as indicated, by one-way analysis of variance (ANOVA), followed by Tukey’s *post-hoc* test. Error bars represent standard error of the mean (SEM). Null hypotheses were rejected at the 0.05 level.

## Results

### Clinical Findings

**Figure 1:**
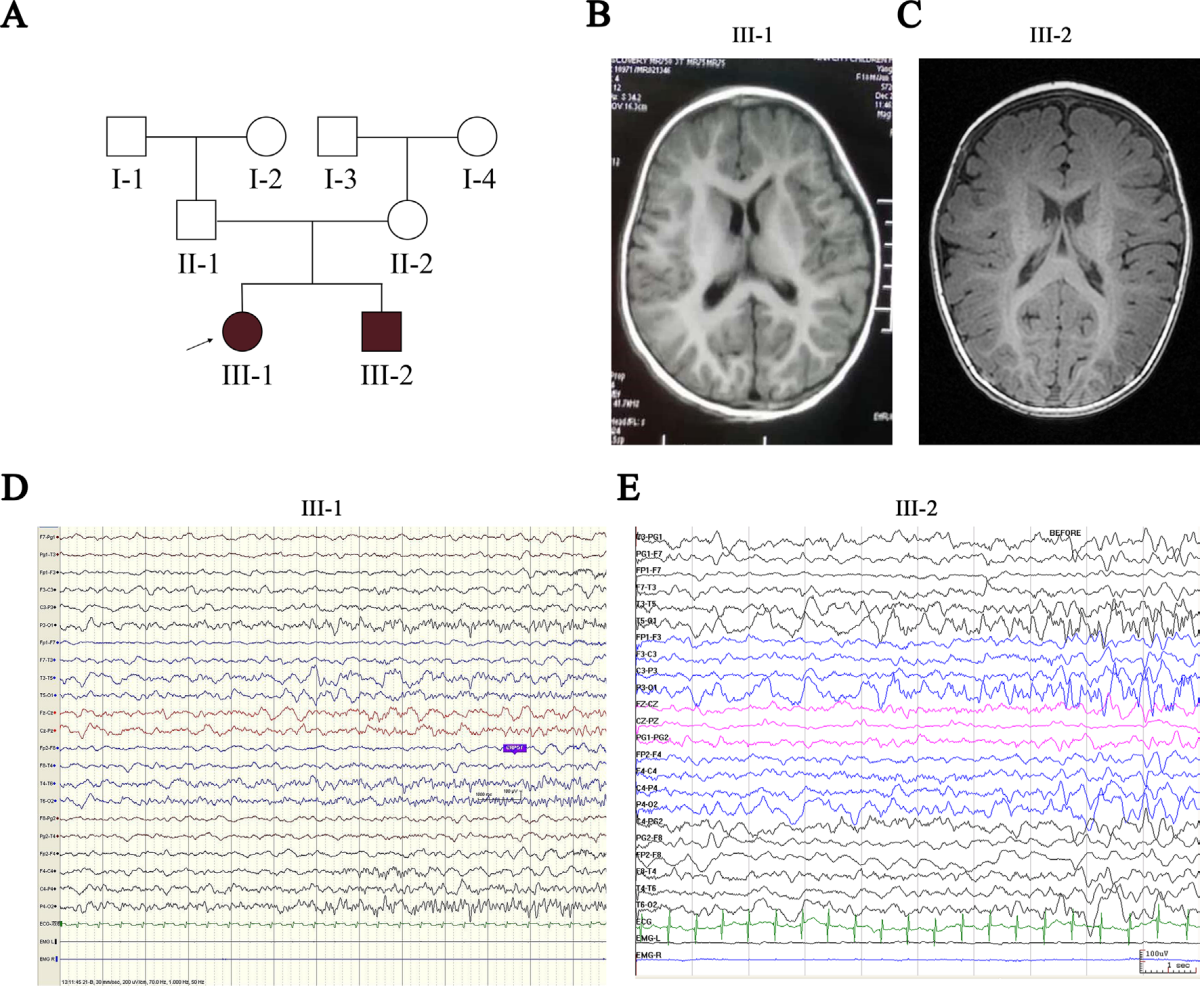
Pedigree of the proband’s family and clinical findings in III-1 and III-2. **(A)** Shaded shapes represent affected family members. The arrow indicates the proband. Squares and circles represent males and females, respectively. **(B, C)** MRI of III-1 and III-2 shows no abnormalities. **(D)** Ictal EEG of III-1 shows slow wave starting from the left temporo-occipital region. **(E)** Ictal EEG of III-2 shows slow wave starting from the bilateral temporo-occipital region. MRI, magnetic resonance imaging; EEG, electroencephalogram.

Patients III-1 and III-2 are siblings born from healthy nonconsanguineous Chinese parents (**Figure 1A**). Patient III-1 initially presented with seizures at the age of 7 months. She suffered from five seizures in the first month and then received antiepileptic drugs (AEDs). Levetiracetam (LEV) was not effective, but oxcarbazepine (OXC) monotherapy has controlled her seizures for more than 2 years, except one status epilepticus incident induced by a common cold (**Table 1**). Neurological examination was normal, and the neuropsychological development examination table for children aged 0∼6 years showed that the full developmental quotient (DQ) value was 93 (gross motor, 90; fine motor, 82; adaptability, 94; language, 94; social behavior, 107), in which the fine motor DQ score was lower than the normal (≧85). Audiological testing using an electroaudiometer showed no hearing impairments, with a hearing threshold of less than 40 dB. However, in auditory brainstem response (ABR) test, patient III-1 displayed slightly prolonged latent phase in III (4.033 ms) and V (6.316 ms) wave for the left ear but not for the right ear, indicating subtle hearing impairment. Blood biochemical tests showed that serum level of potassium was 4.25 μmol/L and magnesium was 0.86 μmol/L, which were all normal. Brain magnetic resonance imaging (MRI) was normal (**Figure 1B**). The first two long-term scalp video electroencephalograms (EEGs) in the first month revealed no abnormalities. The third EEG monitoring captured one clinical seizure, manifested as awake and dialeptic → upper limbs tonic, prominent on right side → eyes deviating to left → generalized tonic-clonic. The ictal EEG showed a slow wave starting from the left temporo-occipital region (**Figure 1D**), and the interictal EEG showed a rare sharp wave over the bilateral parieto-occipito-posttemporal region.

**Table 1:** Characteristics of the affected individuals.

Variable	III-1	III-2
Gender	Female	Male
Age at presentation (months)	7	6
Age at last follow-up (months)	36	20
Seizures	Tonic-clonic	Tonic-clonic
Hearing	Normal	Normal
Fine motor	Delay	Delay
Antiepileptic drug	Oxcarbazepine	Valproic acid
Serum potassium and magnesium	Normal	Normal

Patient III-2 was allergic to OXC, and LEV was ineffective for him. His seizures were controlled by valproate (VPA) for more than 1 year until the present time (**Table 1**). Neurological examination was also normal, and a neuropsychological development examination table for children aged 0∼6 years revealed a DQ value of 83 (gross motor, 93; fine motor, 60; adaptability, 87; language, 93; social behavior, 80), in which the fine motor DQ score was lower than normal. Audiological testing using the electroaudiometer showed no hearing impairments, with a hearing threshold of less than 30 dB. Patient III-2 failed to complete the ABR test because of his noncompliance. The blood biochemical test showed that the serum level of potassium was 4.68 µmol/L and that magnesium was 0.92 µmol/L, both of which were within normal intervals. A brain MRI was also normal (**Figure 1C**). The first two long-term scalp video EEGs in the first 3 weeks revealed no abnormalities as well. The third EEG captured two clinical seizures, manifested as awake and dialeptic → upper limbs tonic → eyes deviating to right → generalized tonic-clonic. The ictal EEG showed a slow wave starting from the bilateral temporo-occipital region (**Figure 1E**), prominent on the right side. There was no interictal discharge.

### Genetic Analysis and Functional Prediction of Kir4.1 Mutations

**Figure 2:**
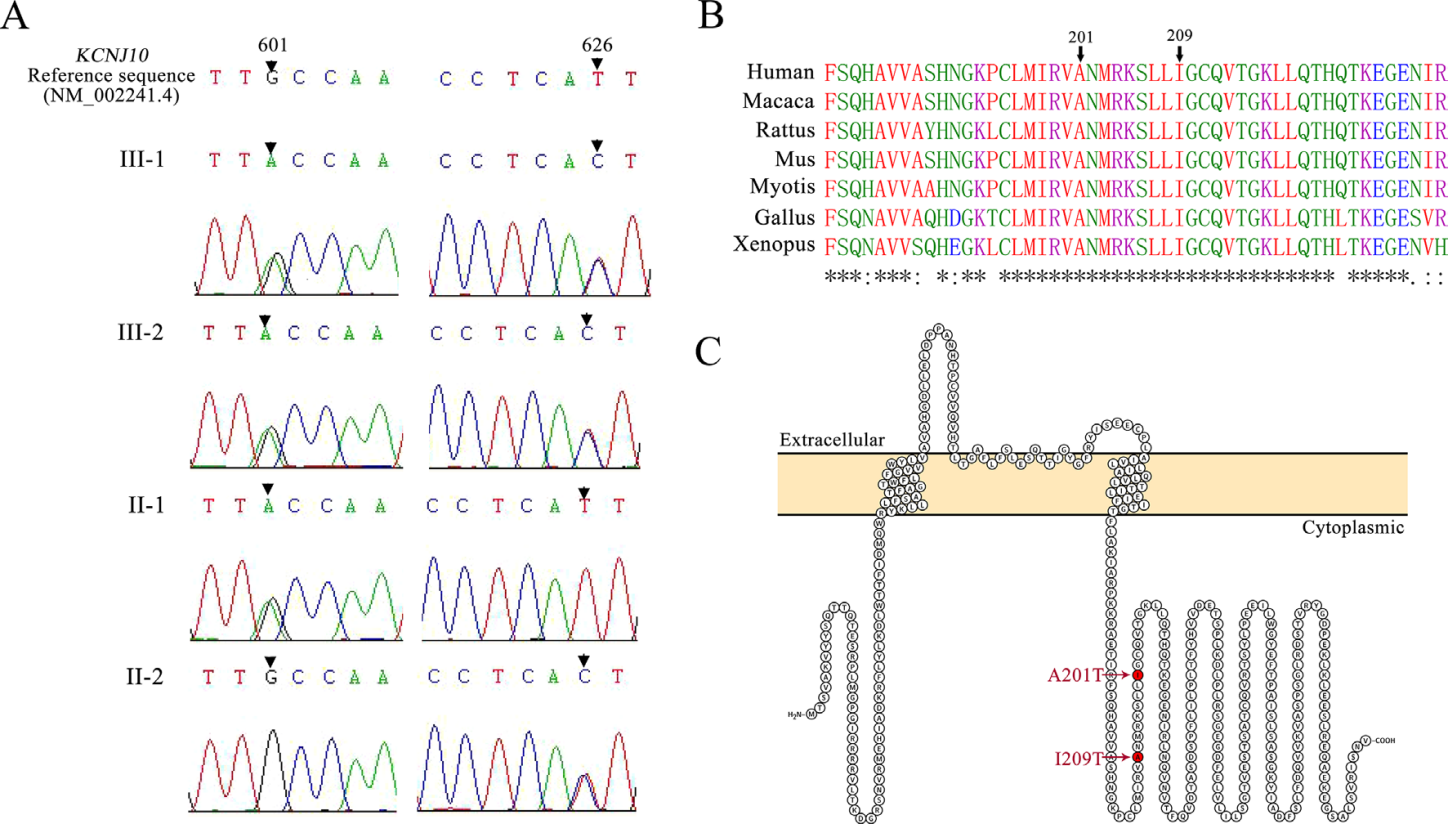
**(A)** Mutation detection by sequencing. Patients III-1 and III-2 are both compound heterozygous for a *601G > A* mutation and a *626T > C* mutation in *KCNJ10*. Their father (II-1) carries a *601G > A* missense mutation, and their mother (II-2) carries a *626T > C *missense mutation. **(B)** Protein alignment for the p. A201T and p.I209T missense mutations using Clustal Omega. Asterisks (*), colons (:), and periods (.), respectively, represent invariant, conserved, and semiconserved residues. **(C)** Protein topology model of KCNJ10 with two transmembrane domains. Both mutations reside in the cytoplasmic domain near the C terminus and are indicated by the red marks.

Patients III-1 and III-2 both have the same compound heterozygous missense mutations in *KCNJ10*, c.601G > A (p.A201T) and c.626T > C (p.I209T). Their unaffected father and mother were heterozygous for c.601G > A and c.626T > C, respectively (**Figure 2A**). The two mutations are not present in ClinVar or in the ExAC database of 60,706 control individuals. A201 and I209 are located in the cytoplasmic C terminus of Kir4.1 (**Figure 2C**) and are conserved among vertebrate species, including mammals, *Gallus* and *Xenopus*, as evaluated by Clustal Omega (**Figure 2B**). To assess the impact of A201T or I209T mutation on Kir4.1 function, analysis tools including PolyPhen-2, PROVEAN, SIFT, and MutationTaster were used. The prediction results uniformly showed that both mutations were deleterious or disease causing (**Table 2**), indicating that A201T and I209T are probably pathogenic mutations.

**Table 2:** Evaluation of possible impact of p.A201T and p.I209T substitutions on Kir4.1 function using online tools.

Tools Mutations	PolyPhen-2		PROVEAN		SIFT		Mutation taster	
**Prediction**	**Score**	**Prediction**	**Score ** **(cutoff = −2.5)**	**Prediction**	**Score** **(cutoff = 0.05**)	**Prediction**	**Probability**
p. A201T	Probably damaging	1.000	Deleterious	−2.894	Deleterious	0.000	Disease causing	0.999
p. I209T	Probably damaging	0.965	Deleterious	−3.886	Deleterious	0.000	Disease causing	0.999

Kir4.1, inwardly rectifying K^+^ channel 4.1.

### Functional Characterization of Kir4.1 Harboring A201T or I209T Mutation

**Figure 3:**
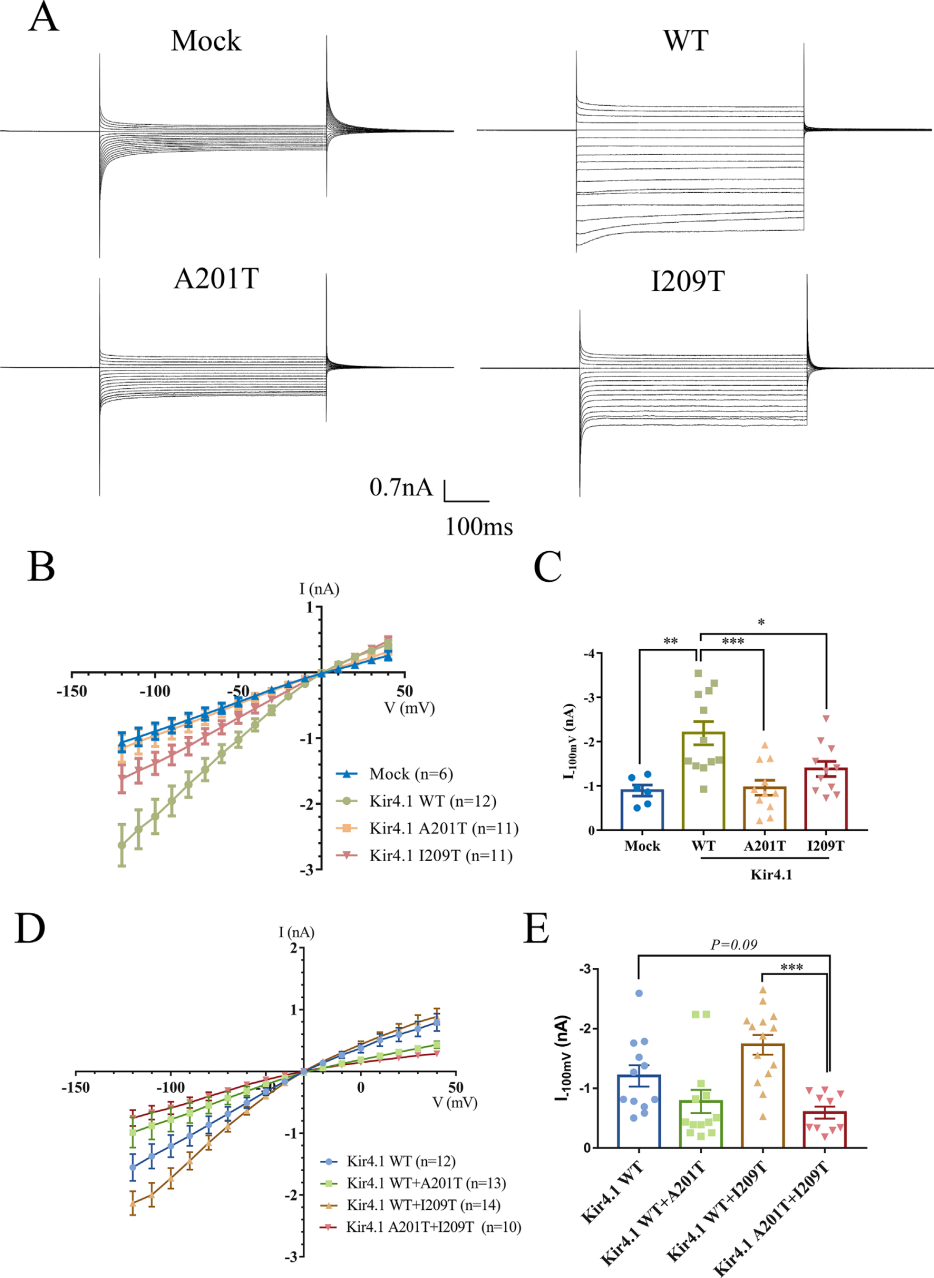
**(A)** Representative current traces in the range of −120 to 40 mV from mock-transfected cells and cells expressing WT and mutant Kir4.1. **(B)** Current–voltage relationship plots from −120 to 40 mV in 10-mV steps. **(C)** Plot of current densities recorded at −100 mV from mock-, Kir4.1 WT-, A201T-, or I209T-transfected cells. **(D)** Current–voltage relationship plots from −120 to 40 mV in 10-mV steps from cells co-expressing WT and mutant Kir4.1. **(E)** Plot of current densities recorded at −100 mV from WT and mutant Kir4.1 co-transfected cells. Data in **(B**–**D)** are presented as the mean ± SEM. *P < 0.05, **P < 0.01, *** *P* < 0.001. WT, wild type; Kir4.1, inwardly rectifying K^+^ channel 4.1.

To evaluate the impact of *KCNJ10* mutations on channel-dependent activity, we conducted whole-cell patch clamp recordings from CHO cells expressing WT or mutant Kir4.1 channels. Surprisingly, currents recorded from A201T-expressing cells were almost identical to mock-transfected cells and were much lower than those from Kir4.1 WT-expressing cells (**Figures 3A, B**). Cells expressing I209T channels also had decreased currents than had cells expressing Kir4.1 WT channels (**Figures 3A, B**). As expected, cells co-expressing Kir4.1 A201T plus I209T also exhibited lower K^+^ current than did cells co-expressing Kir4.1 WT or WT plus I209T (**Figure 3D**). On average, the currents recorded at −100 mV from A201T-expressing cells were −0.96 ± 0.17 nA, which were comparable with those from mock-transfected cells (−0.89 ± 0.12 nA, *P* = 0.99) and significantly lower than those from Kir4.1 WT-expressing cells (−2.19 ± 0.26 nA, *P* < 0.001) (**Figure 3C**). For cells expressing the I209T channel, the mean current recorded at −100 mV was −1.38 ± 0.17 nA, which was also lower than that from cells expressing Kir4.1 WT channel (*P* < 0.05) (**Figure 3C**). K^+^ current in cells transfected with both Kir4.1 A201T and I209T is decreased compared with that from cells transfected with Kir4.1 WT or WT plus I209T (**Figure 3E**). These data indicated that A201T and I209T compound heterozygous mutations compromised Kir4.1 channel function.

### Altered Total and Cell Surface Kir4.1 Levels in A201T- or I209T-Expressing Cells

**Figure 4:**
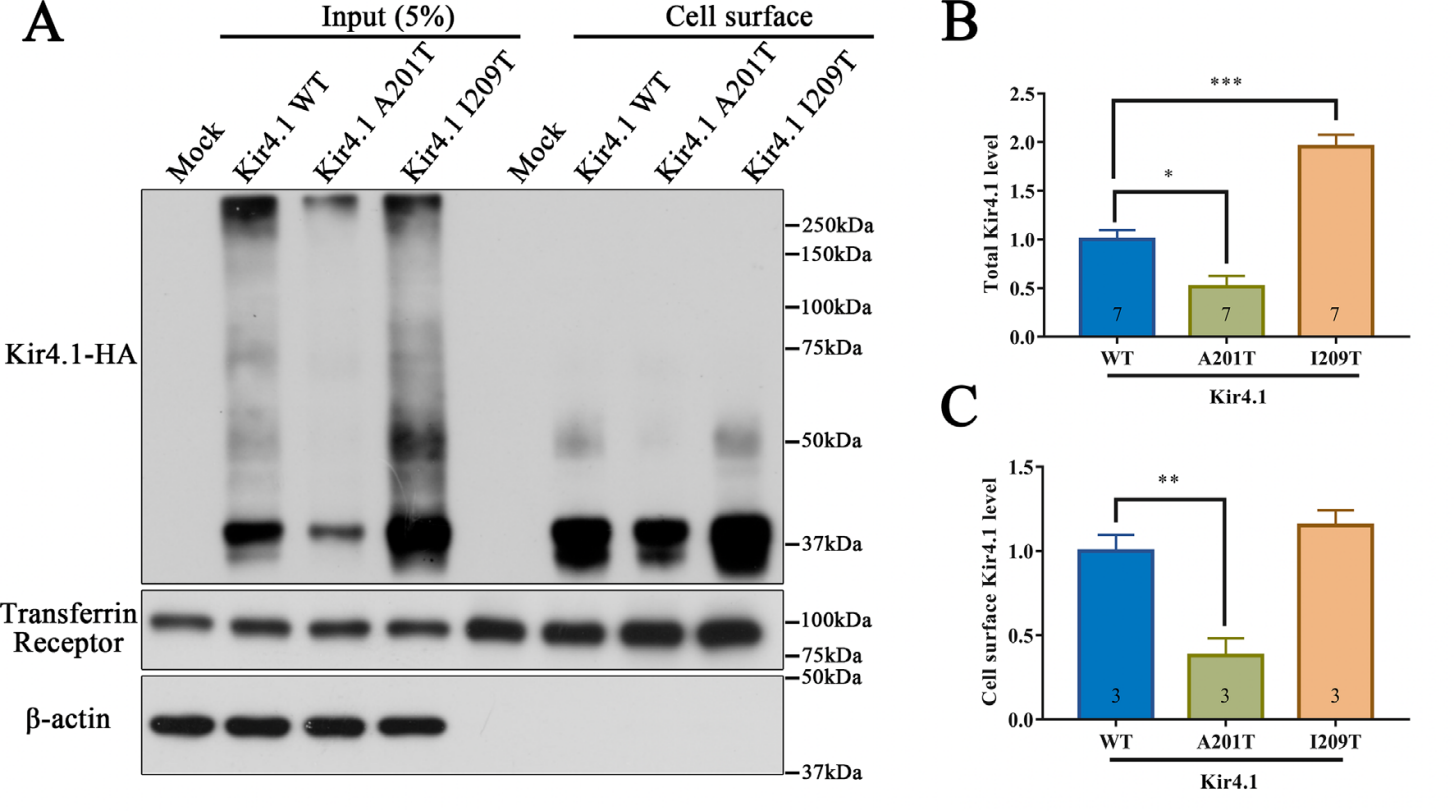
Western blot analysis of Kir4.1 expression using total cell proteins (input) and enriched plasma membrane proteins by biotinylation assay (cell surface) in mock-transfected cells and in cells expressing WT or mutant Kir4.1 channels. **(A)** Representative western blots after biotinylation. The WT and mutant Kir4.1 channels were detected by anti-HA antibody, and β-actin and transferrin receptor served as loading controls for total and cell surface proteins, respectively. **(B, C)** Quantitative analysis of total and cell surface Kir4.1 levels. Data in **(B)** and **(C)** are presented as the mean ± SEM. * P < 0.05, ** *P* < 0.01, *** *P* < 0.001. Kir4.1, inwardly rectifying K^+^ channel 4.1; WT, wild type; HA, human influenza hemagglutinin.

As a membrane protein, Kir4.1 cell surface distribution directly affects its inward rectifying ability. Therefore, we hypothesized that the A201T or I209T mutation disrupts Kir4.1 function possibly *via* altering its cell surface distribution. To test this hypothesis, biotinylation and western blot were carried out to detect cell surface level of Kir4.1, and transmembrane protein transferrin receptor (TfR) was used as a loading control. Interestingly, we observed that both mutations altered total Kir4.1 levels, but in opposite directions, with A201T decreasing and I209T increasing channel expression (**Figures 4A, B**). The cell surface level of Kir4.1 channels was also significantly decreased in cells expressing A201T channels but showed no difference in cells expressing I209T channels (**Figures 4A, C**).

### A201T Mutation Affects Kir4.1 Stability

**Figure 5:**
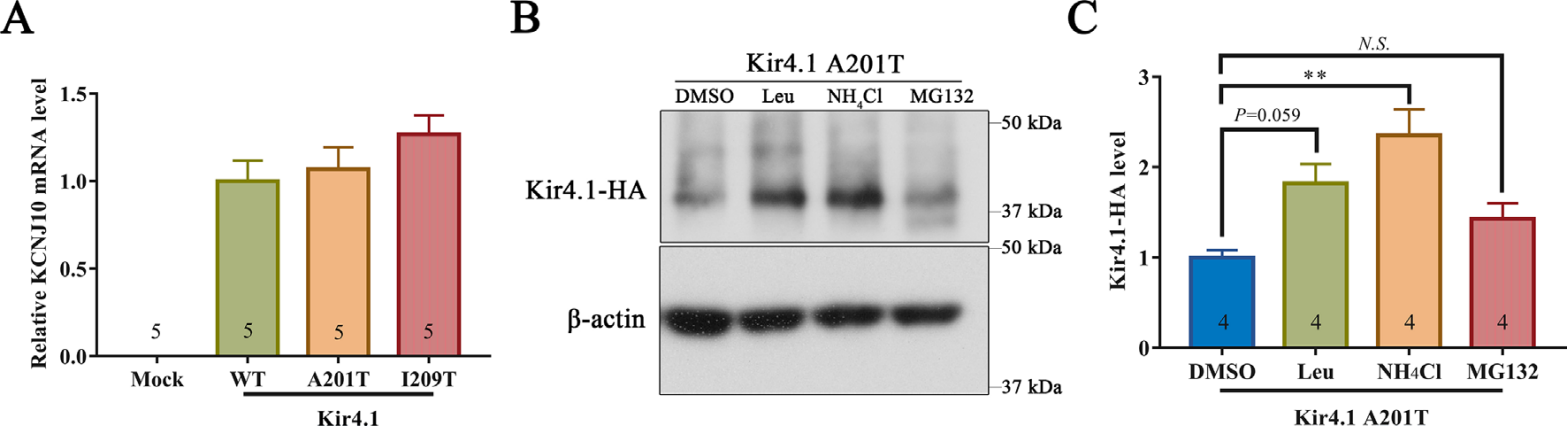
**(A)** *KCNJ10* mRNA levels in 293T cells transfected with different plasmids. **(B, C)** Representative western blots and quantitative analysis of 293T cell lysis probed for anti-HA and β-actin antibodies. After transfection with A201T Kir4.1 plasmid, 293T cells were incubated with 0.1% DMSO, lysosomal inhibitor (Leu or NH_4_Cl) or proteasomal inhibitor MG132. HA, human influenza hemagglutinin; DMSO, dimethylsulfoxide; Leu, leupeptin; *N.S.*, not significant. Data are presented as the mean ± SEM. ** *P* < 0.01.

293T cells expressing mutant and WT Kir4.1 channels showed comparable Kir4.1 mRNA levels, indicating that both A201T and I209T mutations have no impact on the transcription of Kir4.1 (**Figure 5A**). Furthermore, treatment of 293T cells with the lysosomal inhibitor leupeptin or NH_4_Cl largely prevented the degradation of Kir4.1 caused by the A201T mutation (**Figures 5B, C**). However, treatment with the proteasomal inhibitor MG132 showed no such effect (**Figures 5B, C**). These findings suggested that the Kir4.1 protein becomes unstable with the A201T mutation and is more easily degraded through the lysosomal degradation pathway.

## Discussion

Here, we identified two novel variants of *KCNJ10* in a compound heterozygous state in two siblings with SeSAME/EAST-like syndrome. Functional analysis using whole-cell patch clamp recordings showed that both mutations impaired K^+^ channel function but to different degrees. The A201T variation almost abolished the inward K^+^ current, while the I209T variation partly decreased the current. Disruption of Kir4.1 function caused by A201T may, at least in part, result from lower total and cell surface levels of Kir4.1. The A201T mutation did not affect *KCNJ10* gene expression but had an impact on protein stability. Kir4.1 with A201T is more easily degraded in a lysosome-dependent manner.

The typical SeSAME/EAST syndrome consists of four prominent features: epilepsy, ataxia, sensorineural deafness, and renal tubulopathy (Bockenhauer et al., 2009; Scholl et al., 2009). However, the two affected siblings described in this study exhibited only infantile onset of seizures and delayed fine motor ability, with no apparent hearing impairment or electrolyte disturbances. Our findings are in accordance with a recent study in which three siblings with a homozygous Kir4.1 mutation (I60T) were reported to present with seizures, ataxia, and no electrolyte or hearing abnormalities (Al Dhaibani et al., 2018). Similarly, in Jack Russell Terriers, naturally occurring Kir4.1 variants (I209M and L329P) cause marked spinocerebellar ataxia and epilepsy without any effect on electrolyte balance or hearing (Rohdin et al., 2015; Van Poucke et al., 2017). Indeed, the extent of sensorineural deafness in SeSAME/EAST syndrome is variable and can sometimes be absent or identified only with specific testing (Scholl et al., 2012; Cross et al., 2013). Serum electrolyte abnormalities in SeSAME/EAST patients are typically reported to be absent before the age of 3 years, exhibiting progressive worsening with age and becoming abnormal after the age of 5 (Scholl et al., 2012; Cross et al., 2013). Therefore, the normal serum electrolyte levels in the present cases are probably due to their younger ages (less than 3 years old).

For the first time, we identified an Ile to Thr substitution at position 209 of human Kir4.1. Interestingly, amino acid replacement at the same position was also found in Terrier breeds (Rohdin et al., 2015; Gilliam et al., 2014), although the substitution is Ile to Met (I209M) not Ile to Thr. Kir4.1 I209M homozygous mutation in several Terrier breeds leads to SeSAME/EAST-like phenotypes, including spinocerebellar ataxia and myokymia, seizures, or both (Rohdin et al., 2015; Gilliam et al., 2014). It should be noted that Met and Thr are similar with respect to amino acid properties, as both are polar, uncharged, and hydrophilic. Based on this evidence, we speculate that I209T and I209M variants may cause similar pathological consequences, providing additional evidence for I209T as a SeSAME/EAST-like syndrome-causing mutation.

The Kir4.1-mediated K^+^ current was partly compromised by I209T, although the cell surface Kir4.1 level is normal (**Figure 4**). Indeed, the compromised function of Kir4.1 is not always associated with lower cell surface levels. For example, the disease-causing mutation C140R severely disrupted Kir4.1-associated K^+^ conductance but was accompanied by an increase in total and cell surface levels (Sala-Rabanal et al., 2010). We hypothesize that I209 residue neighbors the inferred phosphatidylinositol 4,5-bisphosphate (PIP_2_) binding sites of Kir4.1 (R204, K205, and K216) and may allosterically decrease channel–PIP_2_ interaction (Lopes et al., 2002), which is crucial for channel activity and regulation. Therefore, we inferred that I209T may weaken the interaction between Kir4.1 and PIP2 and thus lead to loss of function.

In summary, we identified novel Kir4.1 compound heterozygous mutations responsible for SeSAME/EAST-like syndrome. Functional analysis showed that A201T abolished and I209T reduced the channel activity. Mincing compound heterozygous variants *via* co-expression of A201T and I209T also disrupted K^+^ channel function. Decreased total and cell surface Kir4.1 levels caused by protein instability likely contribute to the loss of function with A201T mutation. In contrast, I209T does not reduce Kir4.1 level but may weaken the interaction with PIP2 and thus lead to a decrease in channel activity. Further animal studies, such as generation of transgenic mice, are necessary to confirm loss of function of mutant Kir4.1 channels* in vivo*.

## Data Availability Statement

The datasets generated for this study can be found in the NCBI ClinVar database accessions SCV000996517 and SCV000996518.

## Ethics Statement

This study was carried out in accordance with the recommendations of ethical guidelines, Medical Ethics Committee of the Medical School of Xiamen University. This research protocol was approved by the Medical Ethics Committee of the Medical School of Xiamen University. Written informed consents of the children were obtained from their parents for the gene sequencing the publication of this case report, in accordance with the Declaration of Helsinki.

## Author Contributions

HZ, FW, XW, and YZ wrote the manuscript; HZ, RW, YH, YC, and BZ conducted molecular analysis; LZ, RW, YG, and HS performed electrophysiology; FW and YY provided clinical data and conducted genetic analysis; HL and XZ edited the manuscript.

## Funding

This work was supported in part by the National Natural Science Foundation of China (81571176, 31871077, and 81822014 to XW; 81802823 to YZ; 81701349 to HZ; and 81470060 to HS), the National Key R&D Program of China (2016YFC1305900 to XW), China Postdoctoral Science Foundation (2016M600502 to HZ), the Natural Science Foundation of Fujian Province of China (2017J06021 to XW and 2018J01054 to YZ), the Education and Research Foundation for Young Scholars of Education Department of Fujian Province, China (JAT170004 to YZ), and the Fundamental Research Funds for the Chinese Central Universities (20720150061 to XW).

## Conflict of Interest

The authors declare that the research was conducted in the absence of any commercial or financial relationships that could be construed as a potential conflict of interest.
